# Hospital Readmissions Among People With Sickle Cell Disease

**DOI:** 10.1001/jamanetworkopen.2025.17974

**Published:** 2025-06-17

**Authors:** Ruchika Goel, Ping Yang, Xianming Zhu, Eshan U. Patel, Elizabeth P. Crowe, Herleen Rai, Evan M. Bloch, Aaron A. R. Tobian

**Affiliations:** 1 Department of Pathology, Johns Hopkins University School of Medicine, Baltimore, Maryland; 2 Simmons Cancer Institute, Division of Hematology-Oncology, Department of Internal Medicine at SIU School of Medicine, Springfield, Illinois; 3 Department of Epidemiology, Johns Hopkins University Bloomberg School of Public Health, Baltimore, Maryland

## Abstract

**Importance:**

While advances in the management and treatment of sickle cell disease (SCD), the most common inherited disorder in the United States, have improved outcomes, hospital readmissions remain a significant concern. Thirty-day hospital readmissions is a key quality-of-care indicator; however, there are limited contemporary nationally representative data on SCD readmissions.

**Objective:**

To characterize national trends in SCD-related readmissions in the United States and describe factors associated with readmission among patients with SCD.

**Design, Setting, and Participants:**

This cohort study included patients with SCD aged 18 years and older. Patients were identified in the Nationwide Readmissions Database (NRD), an all-payer database of US hospitalizations, using *International Statistical Classification of Diseases, Tenth Revision, Clinical Modification (ICD-10-CM) *codes. Data were collected from January 2016 to December 2021 and analyzed from May to September 2024.

**Main Outcomes and Measures:**

Readmission was defined as subsequent admission within 30 days of hospitalization discharge using the Centers for Medicare & Medicaid Services methodology. The 30-day all-cause unplanned readmission risk among adults with SCD was estimated, and the trend in 30-day readmissions among adults with and without SCD was assessed by calendar year from 2016 to 2021. Survey-weighted mixed-effect Poisson regressions were used to identify factors associated with readmission.

**Results:**

From 2016 to 2021, 140 096 807 all-cause index hospitalizations and 592 951 SCD-related index hospitalizations were analyzed. Patients with SCD had a stable readmission risk of approximately 34% (annual range, 32.6%-34.3%), significantly higher per year than the approximately 12% readmission risk among patients without SCD (annual range, 12.0%-12.2%) (*P* < .001). In 2021, there were 92 536 index admissions from 37 410 unique patients with SCD (median [IQR] age, 34 [26-46] years; 22 484 [60.1%] female), with 30 467 readmissions. Younger patients (aged 18-29 years) had the highest readmission risk at 35.1%. In multivariable model among patients with SCD, patients from the highest-income zip codes had lower readmission risk than those from lower-income areas (adjusted risk ratio [aRR], 0.90; 95% CI, 0.84-0.97). Admissions paid by Medicare and Medicaid were associated with higher readmission risk than private insurance (Medicare: aRR, 1.67; 95% CI, 1.56-1.78; Medicaid: aRR,1.53; 95% CI, 1.43-1.63). Vaso-occlusive crises at index admission were associated with higher readmission risk (aRR, 1.31; 95% CI, 1.25-1.37). Fewer readmissions were observed in patients receiving simple (aRR, 0.86; 95% CI, 0.82-0.91) and exchange (aRR, 0.78; 95% CI, 0.61-0.99) red blood cell transfusions.

**Conclusions and Relevance:**

In this cohort study with nationally representative data, patients with SCD had a significantly higher readmission risk than patients without SCD. Preventative, disease-modifying, and curative interventions are needed to reduce readmission risks and improve outcomes for patients with SCD.

## Key Points

**Question:**

What are the nationally representative trends in and factors associated with hospital readmission in patients with sickle cell disease (SCD)?

**Findings:**

In this cohort study that included more than 140 million index hospitalizations, patients with SCD had a significantly higher risk of readmission (34%) than those without SCD (12%). Among 37 410 patients with SCD, younger patients (aged 18-29 years), patients from the lowest income zip code areas, those with Medicare or Medicaid payments, and patients experiencing vaso-occlusive crises at index admission had higher readmission risk, while fewer readmissions were observed in patients with SCD receiving simple or exchange red blood cell transfusions.

**Meaning:**

In this study, SCD was associated with a significant readmission risk nationally, highlighting the need for preventative, disease-modifying, and curative interventions to improve outcomes for patients with SCD.

## Teaser

This cohort study evaluates readmission risk as well as factors associated with readmission among US adults with sickle cell disease.

## Introduction

Sickle cell disease (SCD), the most common inherited hemoglobinopathy, is a chronic and debilitating inherited blood disorder causing a range of complications over the course of a patient’s lifespan.^1^ Hospitalizations and readmissions are frequent among individuals with SCD due to associated complications such as vaso-occlusive crises (VOCs), acute chest syndrome, fever, sepsis and stroke. Hydroxyurea and chronic simple or exchange red blood cell (RBC) transfusions have been the cornerstone for management of SCD.^2^ Clinicians treating patients with SCD had few disease-modifying therapies to prevent or treat the complications of SCD.^3,4^ The US Food and Drug Administration has approved multiple new therapies for SCD, such as L-glutamine, voxelotor, and crizanlizumab that aim at decreasing transfusion dependence and hospitalizations, but some of these treatments have recently been withdrawn due to concerns with safety or lack of efficacy in the clinical setting.^3,5,6,7,8,9,10^ Potentially curative therapies, such as stem cell transplantation, are transforming the management of SCD.^11^ The most groundbreaking development has been gene therapies, which represent a revolutionary addition to the therapeutic landscape of SCD but remain logistically and cost prohibitive.^12,13^

Thirty-day hospital readmissions are widely recognized as a key indicator of the quality of care.^14,15^ The US Centers for Medicare & Medicaid Services (CMS) has focused on reducing the 30-day hospital readmissions as a key quality-of-care indicator through the Hospital Readmissions Reduction Program (HRRP), introduced in 2012.^16,17,18^ While advances in the medical management of SCD have improved clinical outcomes, readmissions among patients remain an enduring challenge.^19,20,21,22^ A retrospective study of adult patients with SCD in California between 2005 and 2014 found that 10% of patients had 1 readmission and 20% experienced at least 2 readmissions within 30 days; male sex assigned at birth and Medicare coverage were associated with higher readmission risk.^20^ A French study in children showed that long-term chronic diseases, including SCD, cystic fibrosis, and diabetes, had high risk of readmission.^21^ Two studies using the 2016 Nationwide Readmissions Database (NRD), demonstrated between 19% and 27% of patients with SCD were readmitted within 30 days.^22,23^

Understanding the health implications of readmissions of patients with SCD is crucial for implementing effective interventions and improving patient outcomes. However, recent nationwide data among patients with SCD in the United States and comparisons with patients without SCD are limited, and no data are available on national trends in readmission across multiple years. This study aims to quantify the contemporary burden of SCD-related readmissions in the United States and characterize factors associated with readmission among patients with SCD, utilizing data from a nationally representative database.

## Methods

### Data Source

We used the January 2016 to December 2021 NRD to perform a trend analysis; however, the primary analysis used the 2021 NRD, as 2021 was the year with the most recent data available at time of analysis. The NRD is the largest nationally representative all-payer readmissions database, developed by the Agency for Healthcare Research and Quality as part of a federal-state-industry partnership for the Healthcare Cost and Utilization Project (HCUP).^24^ The NRD contains discharge-level information on patient demographic characteristics, clinical characteristics, procedures, and costs from a large sample of community hospitals (all nonfederal short-term general and specialty hospitals) across the United States. NRD has been used widely for readmission assessment.^25,26^ The NRD is constructed annually using discharge data from a single calendar year, with unique verified patient linkage numbers to track patients across hospitals within a state. Each record in the NRD represents a single hospitalization. Same-day readmissions to the same hospital and same-day transfers to another hospital are considered a single hospitalization in the NRD. Each admission records up to 40 *International Statistical Classification of Diseases, Tenth Revision, Clinical Modification (ICD-10-CM) *diagnosis codes (I10_DX1 to I10_DX40) and up to 25 *ICD-10 Procedure Coding System* (*ICD-10-PCS*) procedure codes (I10_PR1 to I10_PR25). The first listed diagnosis is the principal diagnosis (I10_DX1), ie, the condition primarily responsible for the patient’s hospital admission.

The NRD was developed using a one-stage clustered design with stratification sampling. Provided by HCUP, poststratification discharge weights were calculated based on hospital characteristics (census region, urban-rural location, teaching status, bed size, and hospital ownership) and patient characteristics (age, sex) to obtain national estimates.^27^ The 2021 NRD included all hospitalizations from 30 US states with approximately 16.8 million unweighted discharges, representing approximately 61% of the total US resident population and 60% of all US hospitalizations.^27^ After weighting, the NRD represents all US community hospitalizations, corresponding to 33.4 million discharges. The NRD methodology states that it is not designed to support regional, state-specific, or hospital-specific readmission analyses.

An institutional review board exemption was granted by the Johns Hopkins University School of Medicine and informed consent was not required, as the NRD database contains deidentified patient information. This report follows the Strengthening the Reporting of Observational Studies in Epidemiology (STROBE) reporting guidelines for cohort studies.

### Study Population

The study population included all adult (age ≥18 years) index (initial) admissions with a diagnosis of SCD in the United States from the 2021 NRD (January 1, 2021 to November 30, 2021). Following the CMS methodology, index admission was defined as the initial admission for patients aged 18 years or older, excluding admissions for primary psychiatric diagnosis, rehabilitation, or cancer treatment. Patients discharged due to death, against medical advice, or admitted in December were also excluded.^28^ Because patient identifiers cannot be linked across years in NRD, patients with an index hospitalization on or after December 1 were excluded. Patients who were admitted with SCD were identified using the *ICD-10-CM* codes (D570x, D571x, D572x, D574x, and D578x) in any diagnosis field (I10_DX1 to I10_DX40).^29^ In the trend analysis, the study populations were the same except the year of NRD.

### Primary Outcome

The primary outcome was a 30-day all-cause unplanned readmission.^28^ Consistent with the CMS, readmissions were defined as subsequent admission in the same or a different hospital within 30 days of hospitalization discharge.^28^ The time to readmission was calculated by subtracting the length of stay of index admission from the time between index admission and the readmission. If a patient had multiple readmissions within 30 days of discharge, only the first unplanned rehospitalization was counted as a readmission for the original index admission. If the first 30-day rehospitalization was planned, then we treated this person as not readmitted. Planned readmissions were defined as nonacute hospitalizations for scheduled procedures or specific types of care, including transplant surgery, maintenance chemotherapy or immunotherapy, and rehabilitation. A hospital stay could serve as both a readmission from a prior admission and the index admission for a subsequent readmission if it met all the inclusion criteria for an index admission. Transfers between 2 hospitals were not counted as a readmission.

### Assessment of Variables

NRD patient-level characteristics of interest included age, sex, median zip code household income, primary payer, and severity of illness. The All Patient Refined Diagnosis Related Groups (APR DRGs) severity provided by HCUP was used to classify clinical severity of illness.^30^ Additionally, we examined hospital-level features including bed size, teaching status, and rural or urban designation. We also identified comorbidities including stroke, acute chest syndrome, and VOCs using *ICD-10-CM* codes across all available diagnosis fields (I10_DX1 to I10_DX40) (eTable 1 in Supplement 1). The *ICD-10-PCS* codes were used to define RBC transfusion and RBC exchange across all procedure fields (I10_PR1 to I10_PR25) (eTable 1 in Supplement 1). NRD does not provide data on patients’ race or ethnicity.

### Statistical Analysis

Baseline characteristics (first index admission) for people with SCD were summarized. The unit of analysis was patient’s index hospitalization. Survey weights provided by HCUP were applied to all analyses, and Taylor series linearization was used to account for the complex survey design to estimate variance. The trend in 30-day readmissions among adults with and without SCD was assessed by calendar year using NRD 2016 to 2021. Our main analysis used the 2021 NRD to describe the 30-day all-cause unplanned readmission risk among adults with SCD. Univariable mixed-effect Poisson regressions were used to determine risk factors for readmission among patients with SCD, and account for within-person correlation. A multivariable mixed-effect Poisson regression model, including covariates of age, sex, zip code-level median household income, primary payer, severity of illness, bed size of hospital, hospital teaching status, and rural and urban designation, was used to evaluate independent risk factors for readmissions. In another multivariable model, we additionally included RBC transfusion and RBC exchange, stroke, acute chest syndrome, and vaso-occlusive crises. Variables were selected based on biological plausibility of being related to readmission. We also identified the 20 most common principal (I10_DX1) diagnoses for index admission and readmission using *ICD-10-CM* codes.

We conducted a sensitivity analysis using data from the 2019 NRD to compare readmission factors before and during the COVID-19 pandemic, accounting for potential changes on SCD index admissions and readmissions due to the pandemic. For patients with inconsistent survey weights due to visiting multiple hospitals or changes in age within 2021 NRD, each observation was treated as a distinct individual to address the lack of hierarchical structure between hospitals and patients. A new study identifier was created by combining the original patient identifier with the hospital identifier and corresponding survey weight to account for these variations.^25^

Data analyses were performed using Stata/MP version 18 (StataCorp) and R version 4.2 (R Foundation for Statistical Computing). Statistical significance was set at *P* < .05, and all tests were 2-tailed.

## Results

**Figure 1.**
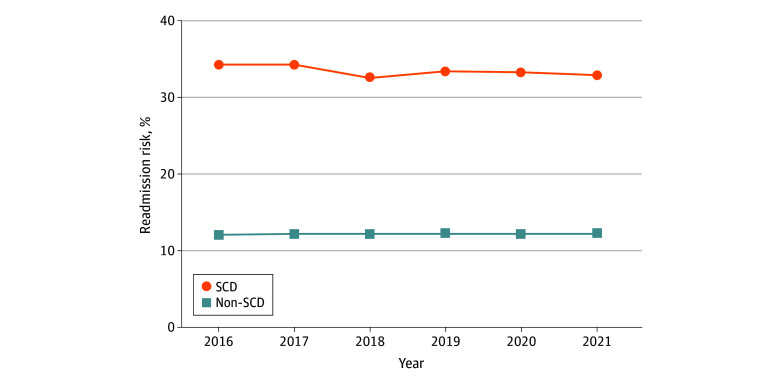
Trends in 30-Day All-Cause Unplanned Readmission Risk for People With and Without Sickle Cell Disease (SCD), 2016-2021 There were 24 242 217 index admissions in 2016 (24 138 568 non-SCD and 103 649 SCD), 24 457 913 in 2017 (24 354 459 non-SCD and 103 454 SCD), 24 352 383 in 2018 (24 251 266 non-SCD and 101 117 SCD), 24 339 493 in 2019 (24 236 784 non-SCD and 102 709 SCD), 21 259 030 in 2020 (21 169 545 non-SCD and 89 486 SCD), and 21 445 771 in 2021 (21 353 235 non-SCD and 92 536 SCD).

From 2016 to 2021, 140 096 807 all-cause index hospitalizations and 592 951 SCD-related index hospitalizations were analyzed. The overall readmission risk for all-cause hospitalizations among patients with SCD remained consistently high at approximately 34% (annual range, 32.6%-34.3%), significantly higher for each individual year than the approximately 12% (annual range, 12.0%-12.2%) for patients without SCD (*P* < .001) (Figure 1).

**Figure 2.**
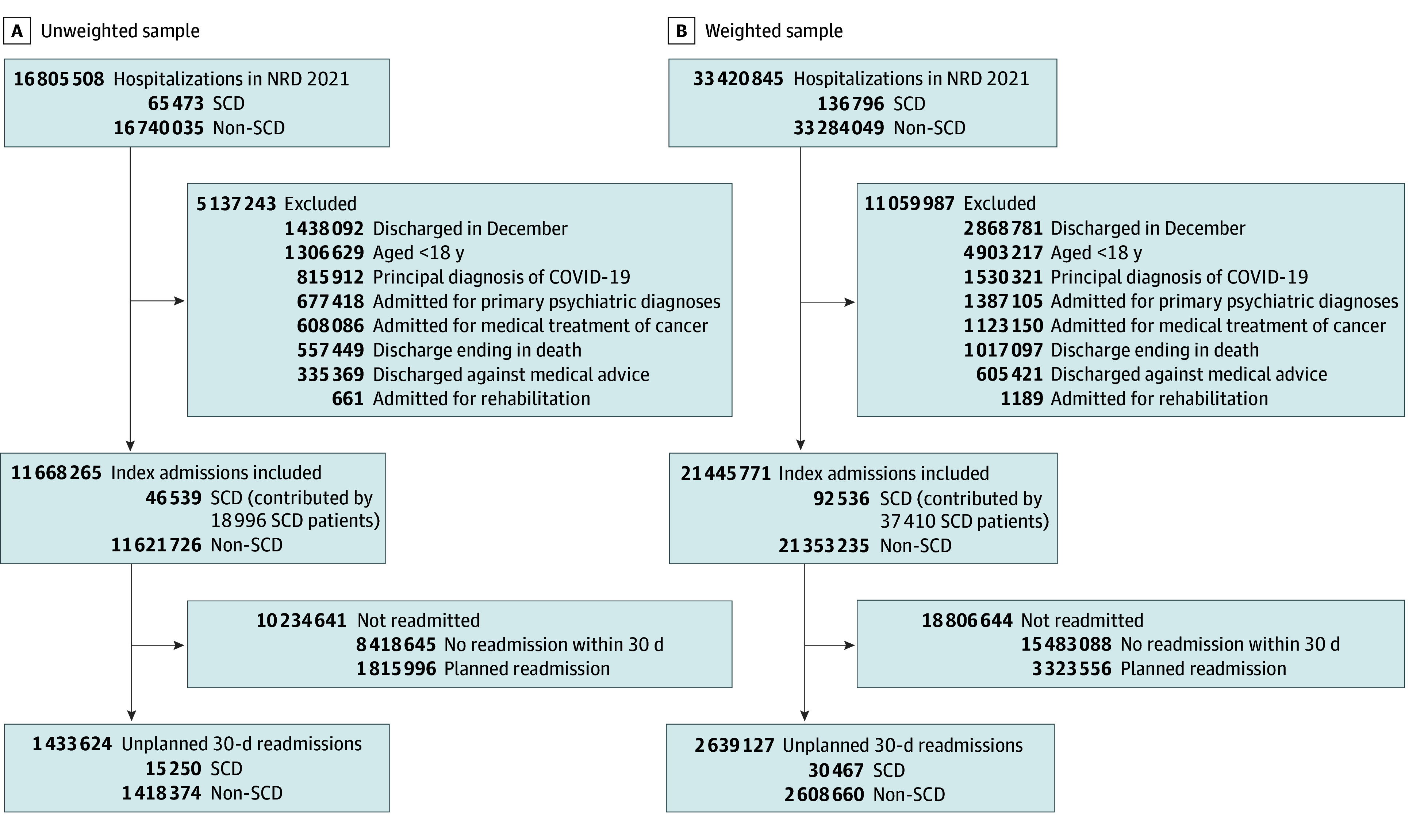
Flowchart of the Index Admissions and Readmissions in 2021 With the Unweighted Sample Sizes and Weighted Population Sizes One person may contribute to 1 or more index hospitalizations in the study population. The subcategories may overlap among the excluded index admissions, so the excluded numbers exceed the total. The analysis follows the 2023 Hospital-Wide All-Cause Unplanned Readmission Measure. NRD indicates Nationwide Readmissions Database; SCD, sickle cell disease.

The 2021 NRD included 16 805 508 hospitalizations (representing 33 420 845 weighted US hospitalizations). Based on our inclusion criteria, we identified 21 445 771 eligible weighted index admissions, with 92 536 index admissions among 37 410 patients with SCD. Of the index admissions, 30 467 (32.9%) had unplanned 30-day readmissions (Figure 2).

Table 1 provides characteristics of the first (baseline) index admission in 2021 for patients with SCD. The median (IQR) age was 34 (26-46) years, and 22 484 (60.1%) were female. Most (70.5%) resided in zip code areas with lower median annual household income (<$52 000, 17 407 [47.0%]; $52 000-$65 999, 8692 [23.5%]), and 26 078 (69.8%) had Medicaid or Medicare coverage. Most SCD admissions occurred in large hospitals (24 218 [64.7%]), teaching hospitals (31 244 [83.5%]), and metropolitan areas (35 742 [95.5%]). More than half of the patients (24 246 [64.8%]) had minor or moderate severity of illness. Among the first (baseline) index admissions for patients with SCD, RBC transfusion was reported in 7830 (20.9%) and RBC exchange transfusion in 201 (0.5%). VOC was the most common diagnosis during hospitalization, reported in 21 481 admissions (57.4%).

**Table 1.** Characteristics of the First Recorded (Baseline) Index Admission of People With SCD in 2021 Nationwide Readmissions Database

Characteristic	Patients, No. (%) (N = 37 410)^a^
Age, y	
18-29	13 735 (36.7)
30-39	10 739 (28.7)
40-49	5442 (14.5)
50-59	3725 (10.0)
60-69	2314 (6.2)
70-79	1019 (2.7)
≥80	435 (1.2)
Sex	
Male	14 926 (39.9)
Female	22 484 (60.1)
Median zip code household income^b^	
$1-$51999	17 407 (47.0)
$52000-$65999	8692 (23.5)
$66000-$87999	6459 (17.4)
≥$88000	4464 (12.1)
Primary payer^c^	
Private insurance	8738 (23.4)
Medicare	11 308 (30.3)
Medicaid	14 770 (39.5)
Self-pay	1580 (4.2)
No charge or other^d^	969 (2.6)
Bed size of hospital	
Small	5668 (15.2)
Medium	7525 (20.1)
Large	24 218 (64.7)
Teaching status of urban hospitals	
Teaching	31 244 (83.5)
Nonteaching^e^	6166 (16.5)
Hospital urban-rural designation	
Metropolitan	35 742 (95.5)
Nonmetropolitan	1667 (4.5)
APR DRG severity of illness	
Minor or moderate loss of function	24 246 (64.8)
Major loss of function	10 104 (27.0)
Extreme loss of function	3040 (8.1)
Red blood cell transfusion	7830 (20.9)
Red blood cell exchange	201 (0.5)
Stroke^f^	530 (1.4)
Acute chest syndrome^f^	2660 (7.1)
Vaso-occlusive crises^f^	21 481 (57.4)

Abbreviations: APR DRG, All Patient Refined Diagnosis Related Groups; SCD, sickle cell disease.

^a^ One person only contributed to 1 admission in this table. Percentages may not add up to 100% because of rounding.

^b^ Data for 1.0% of patients missing.

^c^ Data for 0.2% of patients missing.

^d^ Other payer includes Worker’s Compensation, CHAMPUS, CHAMPVA, Title V, and other government programs.

^e^ Nonteaching includes metropolitan nonteaching and nonmetropolitan hospital.

^f^ Stroke, acute chest syndrome, and vaso-occlusive crises were defined using *International Statistical Classification of Diseases, Tenth Revision, Clinical Modification *codes across all available diagnosis fields (up to 40 diagnostic codes per admission) for the first recorded (baseline) index admission of patients with SCD.

**Figure 3.**
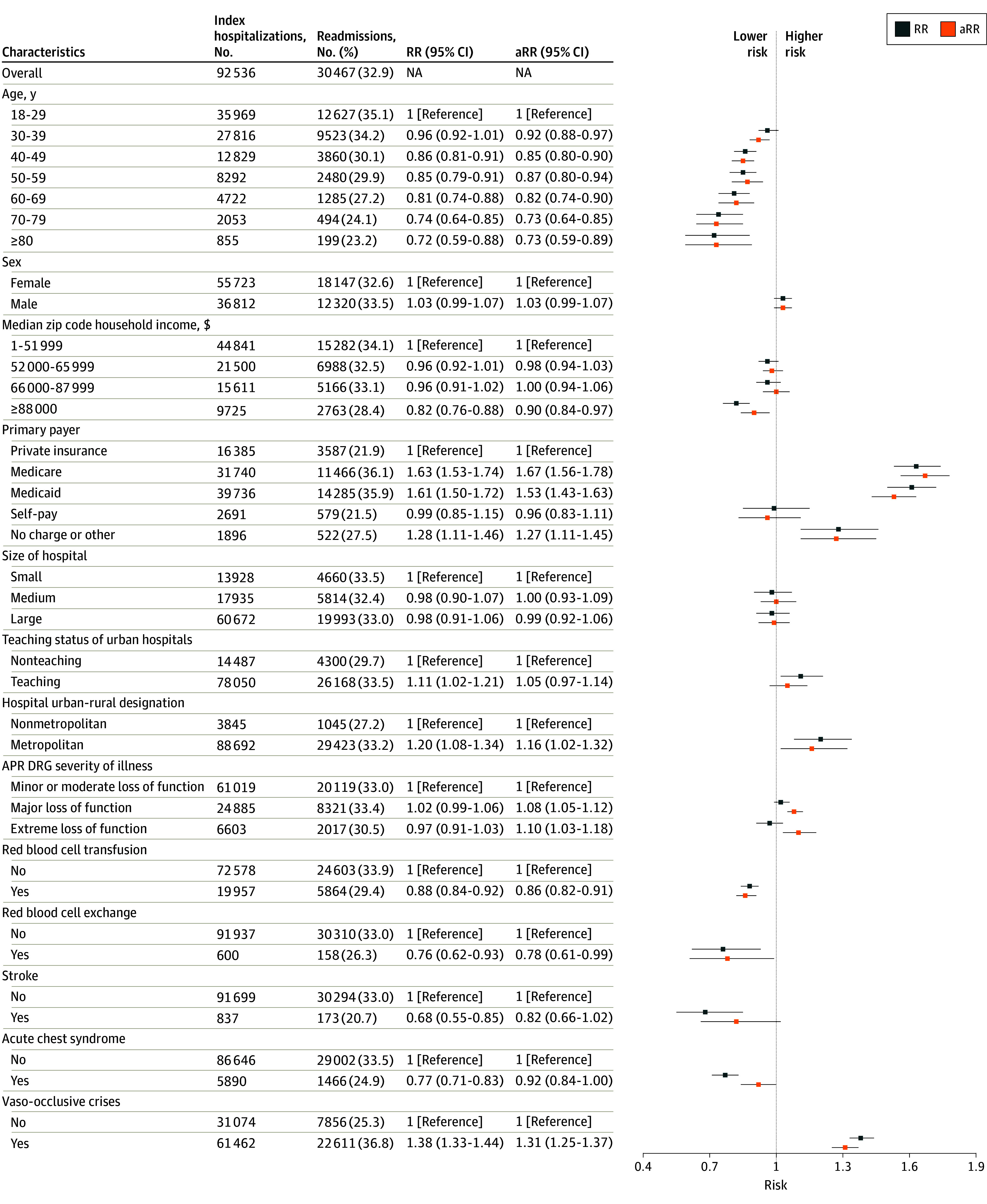
30-Day All-Cause Unplanned Readmission Risk Among People With Sickle Cell Disease in 2021 in the Nationwide Readmissions Database The number and percentage of readmissions are among the index admissions presented. One person may contribute to 1 or more index admissions. Risk ratios (RRs) are from the crude model; adjusted RRs (aRRs) are from multivariable model (ie, multivariable model including all variables in this figure). Both estimates were obtained using mixed-effects Poisson regressions. Other payer includes Worker’s Compensation, CHAMPUS, CHAMPVA, Title V, and other government programs. Nonteaching status includes metropolitan nonteaching and nonmetropolitan hospitals. APR DRG indicates All Patient Refined Diagnosis Related Groups; NA, not applicable.

The associations with all-cause unplanned readmissions among patients with SCD were similar between the univariable and multivariable analyses (Figure 3). Younger individuals with SCD (aged 18-29 years) had the highest crude readmission risk at 35.1%. As patients with SCD aged, crude readmission risks decreased steadily across all age groups to 23.2% for people aged 80 years or older. In the multivariable model, younger individuals with SCD had the highest readmission risk, while the lowest risk was observed in those aged 80 years or older (aRR, 0.73; 95% CI, 0.59-0.89). Admissions from the highest household income zip codes had the lowest risk of readmission than those from lower-income zip codes (aRR, 0.90; 95% CI, 0.84-0.97). Compared with private insurance admissions, higher readmission risks were observed for Medicare (aRR, 1.67; 95% CI, 1.56-1.78) and Medicaid (aRR, 1.53; 95% CI, 1.43-1.63) admissions. Admissions in metropolitan hospitals were associated with higher risks of readmission than nonmetropolitan admissions (aRR, 1.16; 95% CI, 1.02-1.32). Patients with SCD and major or extreme loss of function had greater readmission risks (major loss of function: aRR, 1.08; 95% CI, 1.05-1.12; extreme loss of function: aRR, 1.10; 95% CI, 1.03-1.18). Among clinical factors and interventions, a lower readmission risk was seen among patients with SCD who received either a simple (aRR, 0.86; 95% CI, 0.82-0.91) or exchange (aRR, 0.78; 95% CI, 0.61-0.99) RBC transfusion during the index admissions. VOC was associated with higher risk of readmission (aRR, 1.31; 95% CI, 1.25-1.37) compared with patients admitted without VOC. Overall similar results were seen for 30-day all-cause unplanned readmission risk among people with SCD without adjusting for clinical factors including RBC transfusion, RBC exchange, stroke, acute chest syndrome, and VOC (eFigure 1 in Supplement 1).

Table 2 shows the 20 most common principal diagnoses of index admission. The top 5 causes of index admissions were hemoglobin SS (Hb-SS) disease with crisis, unspecified (51.3%); Hb-SS disease with acute chest syndrome (4.3%); hemoglobin SC disease with crisis, unspecified (3.9%); sepsis, unspecified organism (3.3%); and sickle cell thalassemia, unspecified, with crisis (2.0%). Of note, the readmission risk for patients with SCD admitted for sickle-related conditions was 36.0%, while for those admitted for non–sickle-related conditions was 26.7%. eTable 2 in Supplement 1 presents the 20 most common principal diagnoses for 30-day readmissions.

**Table 2.** Top 20 Principal Diagnoses for Index Admissions Among People With Sickle Cell Disease in 2021 Nationwide Readmissions Database

Principal diagnosis^a^	*ICD-10-CM* code	Patients, No. (%)
Hb-SS disease with crisis, unspecified	D5700	47 422 (51.3)
Hb-SS disease with acute chest syndrome	D5701	3981 (4.3)
Hemoglobin SC disease with crisis, unspecified	D57219	3565 (3.9)
Sepsis, unspecified organism	A419	3049 (3.3)
Sickle cell thalassemia with crisis, unspecified	D57419	1861 (2.0)
Sickle cell disease without crisis	D571	1192 (1.3)
Hb-SS disease with crisis with other specified complication	D5709	939 (1.0)
Sickle cell thalassemia beta plus with crisis, unspecified	D57459	749 (0.8)
Anemia complicating pregnancy, second trimester	O99012	643 (0.7)
Anemia complicating childbirth	O9902	638 (0.7)
Pneumonia, unspecified organism	J189	637 (0.7)
Other specified sepsis	A4189	598 (0.7)
Hypertensive heart and chronic kidney disease with heart failure and stage 1 through stage 4 chronic kidney disease or unspecified chronic kidney disease	I130	553 (0.6)
Sickle cell thalassemia beta zero with crisis, unspecified	D57439	551 (0.6)
Acute kidney failure, unspecified	N179	527 (0.6)
Anemia complicating pregnancy, first trimester	O99011	479 (0.5)
Bloodstream infection due to central venous catheter, initial encounter	T80211A	463 (0.5)
Anemia complicating pregnancy, third trimester	O99013	460 (0.5)
Hypertensive heart disease with heart failure	I110	406 (0.4)
Other sickle cell disorders with crisis, unspecified	D57819	395 (0.4)

Abbreviations: Hb-SS, hemoglobin SS; *ICD-10-CM*, *International Statistical Classification of Diseases, Tenth Revision, Clinical Modification*.

^a^ The principal diagnosis is the first listed diagnosis for all index admissions of people with sickle cell disease. Because this is the first diagnosis for all index admissions, the numbers will not completely correspond to diagnoses listed in Table 1.

In a sensitivity analysis using the pre–COVID-19 pandemic 2019 NRD data, we observed similar results (eFigures 2 and 3 in Supplement 1). However, in 2021, the lowest readmission risk was observed among the oldest adults (aged ≥80 years), while in 2019, those aged 60 to 69 years had the lowest readmission risk. Nonetheless, the readmission risk consistently remained highest in the youngest age group.

## Discussion

Patients hospitalized with SCD have a very high readmission risk compared with those without SCD, thus posing a substantial burden on the US health care system.^22^ These nationally representative data show that on average, a third of all-cause admissions in SCD result in a 30-day readmission compared with 12% overall readmission risk for all other patients with non-SCD diagnoses. Factors that were identified as being associated with higher readmission among patients with SCD in this study included younger patient age (18-29 years), residing in low-income areas, lack of private health insurance, and metropolitan hospital location. Among the factors examined, VOC was the leading clinical diagnosis associated with higher risk of readmission among patients with SCD. Both simple and exchange transfusion were associated with lower risk of hospital readmission in patients with SCD.

Thirty-day hospital readmission is a widely-followed indicator of quality of care with higher risk of readmissions being a surrogate marker of inadequate patient care during the initial hospital stay or suboptimal postdischarge support.^14^ Efforts to reduce 30-day readmissions have become a major focus in health care policy, with hospitals facing financial penalties for high readmission risk.^16,31^ The CMS led HRRP initiatives to decrease unplanned readmissions by incentivizing hospitals to improve discharge planning and postdischarge care coordination.^16,32^ Additionally, disparities in readmission risk highlight the need for targeted strategies to address social determinants of health and ensure equitable care.^31^ The HRRP has led to a notable decline in readmission risk for conditions including heart failure, acute myocardial infarction, and pneumonia^31^; however, it is noteworthy that there has been very little improvement in the risk of readmission for SCD despite the CMS initiatives and advent of multiple novel disease-modifying treatments for SCD, including potential curative treatments.^3,5,6,7,8^ We also did not observe COVID-19 pandemic–related differences in SCD readmissions.

The direct costs of hospitalizations, including inpatient care, medications, and diagnostic tests, contribute significantly to the overall health care burden. Moreover, the indirect costs associated with lost productivity, missed educational opportunities, and reduced quality of life further compound the economic impact of SCD readmissions. A recent cross-sectional study used nationally representative administrative claims data between 2014 to 2021 and demonstrated very low uptake of newly approved disease-modifying treatments despite the approval of newer therapies, including a less than 25% uptake for hydroxurea and a less than 5% uptake for voxelotor and crizanlizumab.^33^ Several cost-effective analyses have evaluated the budget impact of disease-modifying treatments and gene therapies for SCD.^34,35^ The cumulative lifelong care burden for SCD is substantial. According to recent cost-effectiveness analyses, even extremely expensive therapies with curative intent are likely to be cost-effective when considering the lifelong hospital needs—both initial admissions and readmissions and factoring in out-of-hospital care from an equity-informed perspective analysis.^35^

Our study found that the highest readmission risk was among young patients with SCD (aged 18-29 years). Guidelines recommend transfer to adult health care within 6 months of completing pediatric care. However, the transition from pediatric to adult care presents a crucial period during which continuity of care may be disrupted, leading to higher readmission risk and escalated acute resource utilization among young adults.^36,37^

In addition to demographic characteristics, there were several clinical variables associated with readmission. Blood transfusions have previously been reported as a readmission predictor using MarketScan Medicaid Databases, suggesting transfusions as a potential means to reduce the 30-day readmission risk.^38^ Both exchange and simple RBC transfusions, however, were significantly associated with reduced readmissions in SCD in our study. Several out-of-hospital factors potentially contributing to SCD readmissions include inadequate pain management, poor adherence to treatment regimens, lack of access to comprehensive care, and limited availability of specialized SCD centers.^39^ However, our study found that VOC and pain management during the initial hospitalization and social determinants of health, such as insurance status and household income, likely play a substantial role in outcomes like hospital readmission outcomes.

Developing evidence-based guidelines for pain management during VOCs and promoting their implementation in clinical practice can improve patient satisfaction and reduce hospitalization risk.^40^ The economic implications of SCD readmissions requires a multifaceted strategy involving policymakers, health care professionals, and payers. Policy measures should focus on improving access to care, promoting the development of specialized SCD centers, and ensuring adequate reimbursement for comprehensive care services. Collaboration between stakeholders is essential to implement reforms that reduce the financial burden on patients and promote cost-effective and high-quality care delivery.

Utilizing the identified patient-level, sociodemographic, and hospital-level factors associated with hospital readmissions in SCD, derived from the HCUP NRD, a nationally representative database, strategic interventions can be developed to improve care and potentially reduce the burden of both the index hospitalizations and the readmissions for patients with SCD. These interventions may include implementing out-of-hospital and preventative comprehensive care models, enhancing pain management strategies, promoting care coordination, establishing comprehensive SCD clinics, and addressing social determinants of health.

### Strengths and Limitations

This study has strengths. We utilized the largest nationally representative all-payer readmission database in the United States, addressing index as well as readmissions in SCD. There are 30 states contributing data to NRD, but HCUP’s weighting methodology generates nationally representative estimates.

However, there are also limitations of the analyses. The NRD is not designed to support regional, state-specific, or hospital-specific readmission analyses. Readmission in another state cannot be tracked in the NRD because states use different coding for their patient linkage numbers. The patient linkage numbers do not track the same person across years. The hospital identifiers do not track the same hospital across years. Each year of the NRD must be considered a separate sample. Thus, we used the most recent NRD database (2021) to analyze readmissions, and no longitudinal analyses across multiple years were performed except for national trends in readmissions. Furthermore, NRD is an administrative health database and does not have details of clinical variables (eg, laboratory values and temporality of developing comorbidities), which could be associated with readmission.

## Conclusions

In this cohort study, 30-day readmissions were nearly 3 times as common among adults with SCD as adults without SCD. This study, which used the largest nationally representative readmission database, identified various factors associated with hospital readmission in SCD, including patient-level, sociodemographic, and hospital-level characteristics. Addressing the multifactorial causes of initial hospitalizations and any modifiable factors that may affect hospital readmissions is crucial. By identifying these factors, health care stakeholders can develop interventions and policies aimed at reducing readmissions, improving patient outcomes, and optimizing resource allocation in patients with SCD.

## Notes

Supplement 1. **eTable 1.** *ICD-10* Codes Used for the Classification of Procedures and Complications **eTable 2.** Top 20 Principal Diagnoses Leading to 30-Day Readmission Among People With SCD in 2021 NRD **eFigure 1.** Thirty-Day All-Cause Unplanned Readmission Risk Among People With SCD in 2021 NRD **eFigure 2.** Thirty-Day All-Cause Unplanned Readmission Risk Among People With SCD in 2019 NRD **eFigure 3.** Thirty-Day All-Cause Unplanned Readmission Risk Among People With SCD in 2019 NRD

Supplement 2.
Data Sharing Statement
