# Supplementary material for: Hospital Readmissions Among People With Sickle Cell Disease
Source: JAMA Netw Open. 2025 Jun 17;8(6):e2517974. doi: 10.1001/jamanetworkopen.2025.17974 (PMC12175023; doi:10.1001/jamanetworkopen.2025.17974)
Supplement: Supplement 1. — eTable 1. ICD-10 Codes Used for the Classification of Procedures and Complications eTable 2. Top 20 Principal Diagnoses Leading to 30-Day Readmission Among People With SCD in 2021 NRD eFigure 1. Thirty-Day All-Cause Unplanned Readmission Risk Among People With SCD in 2021 NRD eFigure 2. Thirty-Day All-Cause Unplanned Readmission Risk Among People With SCD in 2019 NRD eFigure 3. Thirty-Day All-Cause Unplanned Readmission Risk Among People With SCD in 2019 NRD [file jamanetwopen-e2517974-s001.pdf]

Supplemental Online Content

Goel R, Yang P, Zhu X, et al. Hospital readmissions among people with sickle cell disease. *JAMA Netw Open.* 2025;8(6):e2517974. doi:10.1001/jamanetworkopen.2025.17974

eTable 1. *ICD-10* Codes Used for the Classification of Procedures and Complications

eTable 2. Top 20 Principal Diagnoses Leading to 30-Day Readmission Among People With SCD in 2021 NRD

eFigure 1. 30-Day All-Cause Unplanned Readmission Risk Among People With SCD in 2021 NRD

eFigure 2. Thirty-Day All-Cause Unplanned Readmission Risk Among People With SCD in 2019 NRD

eFigure 3. Thirty-Day All-Cause Unplanned Readmission Risk Among People With SCD in 2019 NRD

This supplemental material has been provided by the authors to give readers additional information about their work.

© 2025 Goel R et al. *JAMA Network Open*.

eTable 1. ***ICD-10*** **Codes Used for the Classification of Procedures and Complications**

**Procedures**	**ICD-10-PCS codes**
Red blood cell transfusion	30233N1, 30233P1, 30243N1, 30243P1, 30253N1, 30253P1, 30263N1, 30230N1, 30230P1, 30240N1, 30240P1
Red blood cell exchange	6A550Z0, 6A551Z0
Bone marrow transplant infusion	30230G4, 30230Y4, 30233G4, 30233Y4, 30240G4, 30240Y4, 30243G4, 30243Y4, 30250G1, 30250Y1, 30253G1, 30253Y1, 30260G1, 30260Y1, 30263G1, 30263Y1
**Comorbidities**	**ICD-10-CM codes**
Stroke	I6300, I63011, I63012, I63013, I63019, I6302, I63031, I63032, I63033, I63039, I6309, I6310, I63111, I63112, I63113, I63119, I6312, I63131, I6319, I6320, I63211, I63212, I63213, I63219, I6322, I63231, I63232, I6329, I6330, I63311, I63312, I63313, I63319, I63321, I63322, I63323, I63332, I63333, I63339, I63341, I63342, I63343, I63349, I6339, I6340, I63413, I63419, I63421, I63422, I63423, I63429, I63431, I63432, I63433, I63442, I63443, I63449, I6349, I6350, I63511, I63512, I63513, I63519, I63523,I63529,I63531, I63532, I63533, I63539, I63541, I63542, I63543, I636, I638, I6381, I6389, I639, I63132, I63133, I63139, I63233, I63239, I63329, I63331, I63411, I63412, I63439, I63441, I63521, I63522, I63549, I6359
Acute chest syndrome	D5701, D57211, D57411, D57431, D57451, D47811
Vaso-occlusive crises	D5741, D57419, D5721, D57219, D570, D5700, D5781, D57819

© 2025 Goel R et al. *JAMA Network Open*.

eTable 2. Top 20 Principal Diagnoses Leading to 30-Day Readmission Among People With SCD in 2021 NRD

Principal diagnosis	ICD-10 code	N (%)
Hb-SS disease with crisis, unspecified	D5700	16829 (55.2)
Sickle-cell/Hb-C disease with crisis, unspecified	D57219	1181 (3.9)
Hb-SS disease with acute chest syndrome	D5701	1067 (3.5)
Sepsis, unspecified organism	A419	866 (2.8)
Sickle-cell thalassemia with crisis, unspecified	D57419	633 (2.1)
Sickle-cell disease without crisis	D571	349 (1.1)
Coronavirus disease-2019 (COVID-19)	U071	339 (1.1)
Hb-SS disease with crisis with other specified complication	D5709	311 (1.0)
Anemia complicating pregnancy, second trimester	O99012	260 (0.9)
Sickle-cell thalassemia beta plus with crisis, unspecified	D57459	251 (0.8)
Hypertensive heart and chronic kidney disease with heart failure and stage 1 through stage 4 chronic kidney disease, or unspecified chronic kidney disease	I130	224 (0.7)
Anemia complicating pregnancy, third trimester	O99013	220 (0.7)
Bloodstream infection due to central venous catheter, initial encounter	T80211A	218 (0.7)
Anemia complicating pregnancy, first trimester	O99011	182 (0.6)
Hypertensive heart and chronic kidney disease with heart failure and with stage 5 chronic kidney disease, or end-stage renal disease	I132	168 (0.6)
Sickle-cell thalassemia beta zero with crisis, unspecified	D57439	151 (0.5)
Other sickle-cell disorders with crisis, unspecified	D57819	147 (0.5)
Pneumonia, unspecified organism	J189	146 (0.5)
Other specified sepsis	A4189	132 (0.4)
Acute kidney failure, unspecified	N179	128 (0.4)

© 2025 Goel R et al. *JAMA Network Open*.

eFigure 1. Thirty-Day All-Cause Unplanned Readmission Risk Among People With SCD in 2021 NRD

Multivariable model without admission diagnosis or hospital treatments.

Abbreviations: RR: risk ratio; SCD: Sickle Cell Disease; APRDRG: All Patient Refined Diagnosis Related Groups; Ref: reference group.

Note: number of readmissions and % readmitted among index admissions presented.
One person may contribute to one or more index admissions.
aRR represented partially multivariable model (i.e. multivariable model including all variables in this figure, but not for RBC transfusion, RBC exchange, stroke, acute chest syndrome or vaso-occlusive crisis). Estimates were obtained using mixed-effects Poisson regressions.

[a] Other payer includes Worker's Compensation, CHAMPUS, CHAMPVA, Title V, and other government programs.

[b] Non-teaching includes Metropolitan non-teaching and Non-metropolitan hospital.

© 2025 Goel R et al. *JAMA Network Open*.

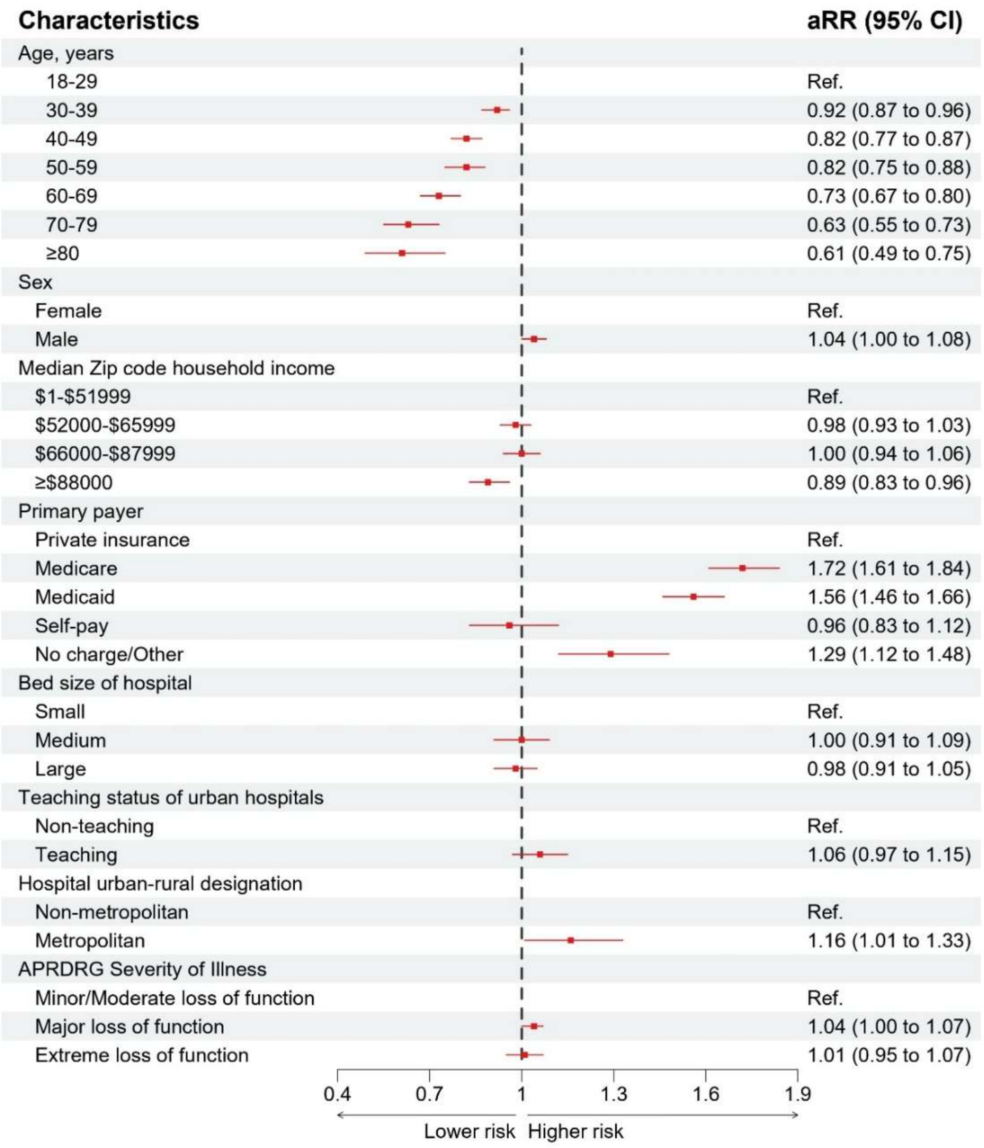

© 2025 Goel R et al. *JAMA Network Open*.

eFigure 2. Thirty-Day All-Cause Unplanned Readmission Risk Among People With SCD in 2019 NRD

Abbreviations: RR: risk ratio; SCD: Sickle Cell Disease; APRDRG: All Patient Refined Diagnosis Related Groups; Ref: reference group.
Note: number of readmissions and % readmitted among index admissions presented.
One person may contribute to one or more index admissions.
RR represented crude model, aRR represented multivariable model (i.e. multivariable model including all variables in this figure). Both estimates were obtained using mixed-effects Poisson regressions.
[a] Other payer includes Worker's Compensation, CHAMPUS, CHAMPVA, Title V, and other government programs.
[b] Non-teaching includes Metropolitan non-teaching and Non-metropolitan hospital.

Characteristics	Index hospitalization	Readmission, n (%)	RR (95% CI)	aRR (95% CI)
Overall	102709	34346 (33.4)		
Age, years				
18-29	41932	14789 (35.3)	Ref.	Ref.
30-39	29253	10265 (35.1)	1.00 (0.96 to 1.04)	0.98 (0.94 to 1.02)
40-49	14494	4458 (30.8)	0.88 (0.83 to 0.93)	0.89 (0.84 to 0.94)
50-59	9209	2713 (29.5)	0.84 (0.78 to 0.90)	0.88 (0.82 to 0.95)
60-69	4708	1199 (25.5)	0.76 (0.70 to 0.82)	0.81 (0.74 to 0.89)
70-79	2098	622 (29.6)	0.90 (0.81 to 0.99)	0.97 (0.86 to 1.09)
≥80	1015	301 (29.6)	0.88 (0.76 to 1.03)	1.00 (0.85 to 1.18)
Sex				
Female	60502	19979 (33.0)	Ref.	Ref.
Male	42206	14367 (34.0)	1.03 (0.99 to 1.08)	1.03 (0.98 to 1.07)
Median Zip code household income				
$1-$47999	51316	17824 (34.7)	Ref.	Ref.
$48000-$60999	22938	7523 (32.8)	0.94 (0.91 to 0.98)	0.95 (0.91 to 0.99)
$61000-$81999	17298	5638 (32.6)	0.94 (0.89 to 0.98)	0.96 (0.92 to 1.01)
≥$82000	9939	2944 (29.6)	0.85 (0.80 to 0.91)	0.92 (0.86 to 0.98)
Primary payer				
Private insurance	18102	4353 (24.0)	Ref.	Ref.
Medicare	35726	12756 (35.7)	1.48 (1.40 to 1.57)	1.48 (1.40 to 1.57)
Medicaid	42534	15706 (36.9)	1.51 (1.42 to 1.60)	1.44 (1.35 to 1.53)
Self-pay	4013	903 (22.5)	0.96 (0.87 to 1.07)	0.94 (0.85 to 1.04)
No charge/Other	2190	577 (26.3)	1.09 (0.94 to 1.26)	1.06 (0.92 to 1.23)
Bed size of hospital				
Small	14817	4937 (33.3)	Ref.	Ref.
Medium	22793	7473 (32.8)	0.98 (0.91 to 1.05)	0.98 (0.91 to 1.06)
Large	65098	21936 (33.7)	1.00 (0.93 to 1.07)	0.99 (0.93 to 1.06)
Teaching status of urban hospitals				
Non-teaching	18561	5886 (31.7)	Ref.	Ref.
Teaching	84147	28460 (33.8)	1.05 (0.99 to 1.11)	0.99 (0.93 to 1.05)
Hospital urban-rural designation				
Non-metropolitan	5285	1522 (28.8)	Ref.	Ref.
Metropolitan	97423	32824 (33.7)	1.15 (1.04 to 1.27)	1.19 (1.07 to 1.34)
APRDRG Severity of Illness				
Minor/Moderate loss of function	72457	24074 (33.2)	Ref.	Ref.
Major loss of function	24958	8630 (34.6)	1.04 (1.01 to 1.08)	1.11 (1.08 to 1.15)
Extreme loss of function	5294	1642 (31.0)	0.96 (0.91 to 1.03)	1.11 (1.04 to 1.18)
Red Blood Cell Transfusion				
No	79937	27448 (34.3)	Ref.	Ref.
Yes	22771	6898 (30.3)	0.89 (0.86 to 0.93)	0.88 (0.85 to 0.91)
Red Blood Cell Exchange				
No	102042	34185 (33.5)	Ref.	Ref.
Yes	666	161 (24.2)	0.72 (0.57 to 0.91)	0.74 (0.58 to 0.94)
Stroke				
No	101818	34198 (33.6)	Ref.	Ref.
Yes	891	148 (16.7)	0.54 (0.44 to 0.67)	0.64 (0.51 to 0.78)
Acute chest syndrome				
No	96238	32743 (34.0)	Ref.	Ref.
Yes	6470	1603 (24.8)	0.76 (0.71 to 0.81)	0.92 (0.86 to 0.99)
Vaso-occlusive Crises				
No	31227	7912 (25.3)	Ref.	Ref.
Yes	71482	26434 (37.0)	1.39 (1.34 to 1.45)	1.36 (1.30 to 1.42)

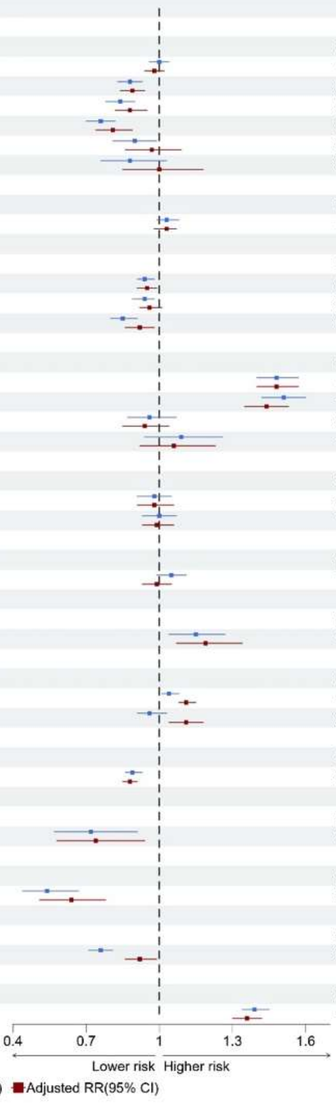

© 2025 Goel R et al. *JAMA Network Open*.

eFigure 3. Thirty-Day All-Cause Unplanned Readmission Risk Among People With SCD in 2019 NRD

Multivariable model without admission diagnosis or hospital treatments.

Abbreviations: RR: risk ratio; SCD: Sickle Cell Disease; APRDRG: All Patient Refined Diagnosis Related Groups; Ref: reference group.

Note: number of readmissions and % readmitted among index admissions presented.

One person may contribute to one or more index admissions.

aRR represented partially multivariable model (i.e. multivariable model including all variables in this figure, but not for RBC transfusion, RBC exchange, stroke, acute chest syndrome or vaso-occlusive crisis). Estimates were obtained using mixed-effects Poisson regressions.

[a] Other payer includes Worker's Compensation, CHAMPUS, CHAMPVA, Title V, and other government programs.

[b] Non-teaching includes Metropolitan non-teaching and Non-metropolitan hospital.

© 2025 Goel R et al. *JAMA Network Open*.

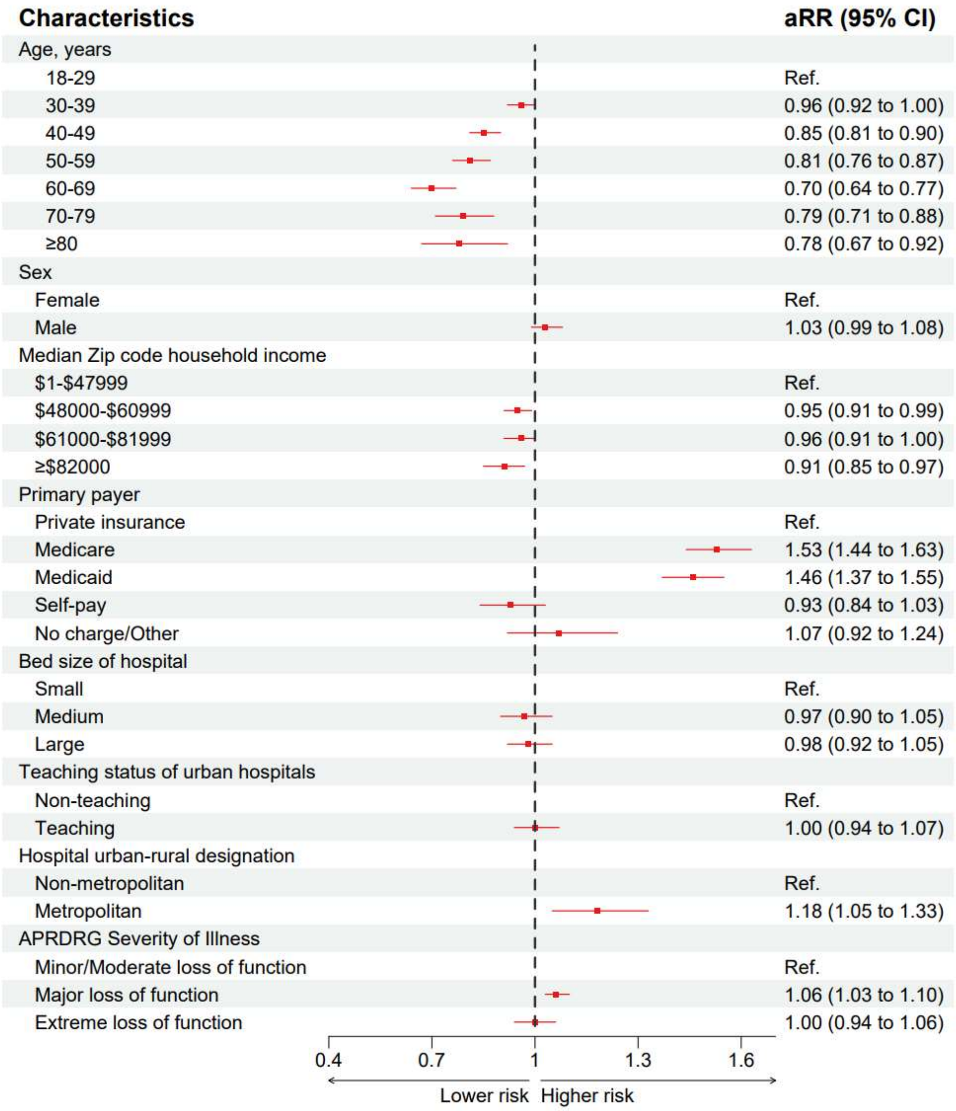

© 2025 Goel R et al. *JAMA Network Open*.
