# Supplementary material for: Hospital Readmissions Among People With Sickle Cell Disease
Source: JAMA Netw Open. 2025 Jun 17;8(6):e2517974. doi: 10.1001/jamanetworkopen.2025.17974 (PMC12175023; doi:10.1001/jamanetworkopen.2025.17974)
Supplement: Supplement 2. — Data Sharing Statement [file jamanetwopen-e2517974-s002.pdf]

Data Sharing Statement

Goel. Hospital Readmissions Among People With Sickle Cell Disease. *JAMA Netw Open*. Published June 17, 2025. doi:10.1001/jamanetworkopen.2025.17974

Data
Data available: Yes
Data types: Deidentified participant data
How to access data: National Readmission Database is already publicly available.
When available: With publication

Supporting Documents
Document types: None

Additional Information
Who can access the data: Anyone requesting the data
Types of analyses: For all purposes
Mechanisms of data availability: Without investigator support
